# Açai Berry Mitigates Parkinson’s Disease Progression Showing Dopaminergic Neuroprotection via Nrf2-HO1 Pathways

**DOI:** 10.1007/s12035-022-02982-5

**Published:** 2022-08-15

**Authors:** Ramona D’Amico, Daniela Impellizzeri, Tiziana Genovese, Roberta Fusco, Alessio Filippo Peritore, Rosalia Crupi, Livia Interdonato, Gianluca Franco, Ylenia Marino, Alessia Arangia, Enrico Gugliandolo, Salvatore Cuzzocrea, Rosanna Di Paola, Rosalba Siracusa, Marika Cordaro

**Affiliations:** 1 Department of Chemical, Biological, Pharmaceutical and Environmental Sciences, University of Messina, Viale Ferdinando Stagno D’Alcontres 31, 98166 Messina, Italy; 2 Department of Clinical and Experimental Medicine, University of Messina, Via Consolare Valeria, 98125 Messina, Italy; 3 Department of Veterinary Sciences, University of Messina, Polo Universitario Dell’Annunziata, 98168 Messina, Italy; 4 Department of Pharmacological and Physiological Science, Saint Louis University School of Medicine, Saint Louis, MO 63104 USA; 5 Department of Biomedical, Dental and Morphological and Functional Imaging, University of Messina, Via Consolare Valeria, 98125 Messina, Italy

**Keywords:** Neurodegeneration, Oxidative stress, Inflammation, Parkinson’s disease, Açai berry

## Abstract

The current pharmacological treatment for Parkinson’s disease (PD) is focused on symptom alleviation rather than disease prevention. In this study, we look at a new strategy to neuroprotection that focuses on nutrition, by a supplementation with Açai berry in an experimental models of PD. Daily orally supplementation with Açai berry dissolved in saline at the dose of 500 mg/kg considerably reduced motor and non-motor symptom and neuronal cell death of the dopaminergic tract induced by 4 injections of 1-methyl-4-phenyl-1,2,3,6-tetrahydropyridine (MPTP). Furthermore, Açai berry administration reduced α-synuclein aggregation in neurons, enhanced tyrosine hydroxylase and dopamine transporter activities, and avoided dopamine depletion. Moreover, Açai berry administration was able to reduce astrogliosis and microgliosis as well as neuronal death. Its beneficial effects could be due to its bioactive phytochemical components that are able to stimulate nuclear factor erythroid 2–related factor 2 (Nrf2) by counteracting the oxidative stress and neuroinflammation that are the basis of this neurodegenerative disease.

## Introduction

Parkinson’s disease (PD) is the second most common neurological illness among those over the age of 65 [1]. The selective loss of dopamine (DA) neurons in the substantia nigra pars compacta (SNpc) and DA levels in the corpus striatum of the nigrostriatal DA pathway in the brain are linked to PD. Because of the loss of DA, the basal ganglia circuitries become dysregulated, resulting in motor symptoms including bradykinesia, resting tremor, stiffness, and postural instability, as well as non-motor symptoms like sleep difficulties, depression, and cognitive deficiencies [2]. The role of oxidative stress in the etiopathology of this illness is widely acknowledged. Increased quantities of oxidized lipids, proteins, and DNA are seen in the SN of PD patients. Furthermore, in PD brains, levels of reduced glutathione (GSH), the most common thiol-reducing agent, are much lower, indicating oxidative stress and nigral degeneration [3]. Chronic neuroinflammation is another key source of ROS in PD patients. Proinflammatory cytokines build up in PD patients’ cerebrospinal fluid and are elevated in postmortem brain samples and experimental models of the illness [4].

Despite advances in our understanding of the pathophysiology of PD, recent research have shown that the Nrf2 (NF-E2-related factor 2)/antioxidant responsive element (ARE) signaling cascade is the most potential target for therapeutic treatment [5].

It is a Cap’n’Collar transcription factor expressed in most brain cell types, including DAergic neurons, astroglia, and microglia, where it contributes to redox homeostasis by regulating the expression of antioxidant genes [6–8]. Several experimental evidence clearly imply that Nrf2 has a role in the neuroprotection of DAergic neurons. In fact, in parkinsonian SN DAergic neurons, Nrf2 translocates to the nucleus, but in normal age-matched controls, it stays cytosolic. This is seen as an attempt to keep ROS production under check [9, 10].

Because oxidative stress play a key role in the majority of instances of Parkinson’s disease, it is critical to comprehend the significance of diet in neuroprotection. Some foods have shown promise in lowering the incidence of Parkinson’s disease in recent epidemiological research [11]. The health advantages linked with the consumption of phytochemicals found in fruits and vegetables result in less functional loss as people age, which may help to halt the onset of Parkinson’s disease [12]. High consumption of fruits, vegetables, and fish was found to be inversely related to the risk of Parkinson’s disease in epidemiological research [13, 14].

Açai seeds have recently piqued the interest of scientists. Açai berry is a berry that has a wide range of nutritional characteristics as well as some medicinal potential. This sour and pleasant-tasting fruit comes from the Euterpe Oleracea palm, which is only found in the Amazon. The Açai fruit, which is considered a high-energy meal, has been used by Amazonian Indians for millennia as a food source and natural cure for a variety of ailments [15–23].

Because of the Açai berry’s high bioactivate nutritional and phytochemical content, its pulp has been widely studied. The composition of Açai berry pulp revealed that it includes a variety of physiologically active phytochemicals as well as large levels of mono- and polyunsaturated fatty acids do not present in most fruits and other berries. Açai pulp contains phytochemicals such as anthocyanins, proanthocyanidins, and other flavonoids. Furthermore, phytochemical tests found that the Açai berry contains various forms of anthocyanins, including cyanidin, delphinidin, malvidin, pelargonidin, and peonidin, as well as a high concentration of luteolin, quercetin, dihydrokaempferol, and chrysoerial, among other polyphenolics. Carotenoids were found in Açai berry pulp in five different forms: carotene, lycopene, astaxanthin, lutein, and zeaxanthin [24].

Açai berry extract and its bioactive content have a wide range of pharmacological effects, including anti-inflammatory, antioxidant, anticarcinogenic, and neuroprotective characteristics, according to a large body of research [25]. However, there is currently a scarcity of scientific data to support the favorable neuroprotective effects. For this reason, we used a consolidated murine model of PD, to investigate the potential beneficial effects of Açai supplementation and the molecular way by which its acts.

## Material and Methods

### Animals

C57/BL6 mice (male 25–30 g, 8 weeks age old; Envigo, Italy) were accommodated in a controlled environment and equipped with standard rodent chow and water. The University of Messina Review Board for animal care (OPBA) approved the study. All animal experiments agree with the new Italian regulations (D.Lgs 2014/26), EU regulations (EU Directive 2010/63), and the ARRIVE guidelines.

### Parkinson Disease Induction

Mice received four intraperitoneal injections of 20 mg/kg of MPTP (Sigma, St. Louis, MO) in saline at 2-h intervals in 1 day, the entire dose per mouse being 80 mg/kg [26].

### Experimental Groups

Mice were indiscriminately distributed to the following groups:

- *Sham* = vehicle solution (saline) was administered intraperitoneally during the 1st day, as for MPTP.
- *Sham* + *Açai* = same as the Sham group, but Açai berry (500 mg/kg) (dissolved in saline) was orally administered starting 24 h after the first vehicle solution injection and continuing through 7 additional days after the last injection of saline (data not shown).
- *MPTP* = MPTP was administered as described above plus administration of saline.
- *MPTP* + *Açai* = but Açai berry (500 mg/kg) (dissolved in saline) was orally administered starting 24 h after the first vehicle solution injection and continuing through 7 additional days after the last injection of saline.

At the conclusion of the experiment, mice were sacrificed under anesthesia and the brain removed and fixed in 10% neutral-buffered formalin or stored at − 70 °C for biochemical and molecular analyses.

### Behavioral Testing

Behavioral assessments on each mouse were made 7 days after MPTP injection. Behavioral data analysis was performed by observers who were unaware of the experimental groups.

- *Pole test (PT)*: The PT was performed as previously described [27]. Briefly, mice are placed with their head upwards right below the top. Two parameters were assessed: time until the animal turned by 180°, and total time until the animal descended to the floor [27].
- *Rotarod test (RT)*: Motor activity was assessed with rotary rod apparatus using a protocol previously described [28, 29]. In brief, after the training sessions, animal was placed back on the drum immediately after falling up to five times in one session.
- *Balance beam walking (BBW)*: The mice were placed to a batten and enticed to cross a timber balancing beam with food [30]. If the mouse slid off, the test was halted and restarted. The time it took a mouse to cross the balancing beam successfully was recorded.
- *Grid walking (GW)*: The grid walking test was used to assess the sensorimotor coordination of mice’s hindlimbs. When a paw totally failed to hold a rung, an independent experimenter tallied the number of hindlimb slides. The average of the foot slips was used for analysis after each experiment was done three times [30].
- *Cylinder test (CiT)*: When mice are maintained in a new transparent cylinder, they investigate by moving around and elevating their bodies to contact the cylinder walls with their forelimbs; this is known as rearing. Before another rearing, we only counted when the mouse elevated both forelimbs above shoulder level and removed both forelimbs from the cylinder [31].
- *Catalepsy test (CaT)*: Catalepsy, demarcated as a reduced capability to start movement and a failure to correct posture, was measured as previously described [32, 33]. In particular, after the training the length of time the mice maintained this position was recorded.
- *Elevated plus-maze test (EPM)*: EPM was performed as previously described [34, 35]. The EPM test was performed to evaluated the anxiety state as described previously [35, 36]. Briefly, after the training session, the number of entries into each arm and the number of crossings were recorded.
- *Open field test (OFT)*: Locomotor activity and anxiety-like behavior were monitored by the OFT. After a training session, each mouse was gently placed in the center of the box, and activity was scored as a line crossing when a mouse removed all four paws from one square and entered another [37, 38].
- *Tail suspension test (TST)*: The tail suspension test is a desperation-based test that measures how long animals remain immobile after being subjected to inexorable conditions. Mice were only considered immobile when they were fully still [39].
- *Forced swimming test (FST)*: The duration of floating (i.e., the time during which the mice made just the modest movements required to keep their heads above water) was scored after each mouse was gently placed in the cylinder for 6 min as previously described [40, 41].
- *Von Frey test (VFT)*: When the paw was inadvertently contacted with von Frey filament, each mouse was watched for paw withdrawal reflex as previously described [42].
- *Tail-flick test (TFT)*: When each mouse’s tail was dipped in a water bath kept at a constant temperature (53 °C), a tail flick response was observed. The experiment was videotaped, and the animal’s reaction time (tail flick) was recorded [42].

### Histology

Brain sections were stained with hematoxylin/eosin (H/E) and studied under light microscopy connected to an imaging system Leica DM6 microscope (Leica Microsystems SpA, Milan, Italy) with Leica LAS X Navigator software (Leica Microsystems SpA). [43]. Histological assessment was made by a blinded observer, and slides were scored for severity of pathological profiles after H/E staining using a semiquantitative 5-point rating scale, as previously described by [43–47].

### Western Blot Analysis of IκBα, GFAP, Iba-1, Nrf2, HO-1, NF-κB p65, Bax, Bcl-2, β-Actin, and Lamin A/C

Western blot analysis was performed as previously described [48–53]. The following primary antibodies were used: IκBα (1–500 Santa Cruz Biotechnology, Heidelberg, Germany #sc1643), glial fibrillary acidic protein (GFAP) (1–500 Santa Cruz Biotechnology, Heidelberg, Germany #sc33673), Iba-1 (1–500 Santa Cruz Biotechnology, Heidelberg, Germany #sc32725), Nrf2 (1–500, Santa Cruz Biotechnology, Heidelberg, Germany, #sc-365949), anti-heme oxygenase 1 (HO-1) (1–500, Santa Cruz Biotechnology, Heidelberg, Germany, #sc-136960), nuclear factor-kappaB (NF-κB) p65 (1–500, Santa Cruz Biotechnology, #sc8414), Bax (1–500 Santa Cruz Biotechnology, Heidelberg, Germany #sc20067), and Bcl-2 anti-Bcl-2 (1–500, Santa Cruz Biotechnology, Heidelberg, Germany, #sc7382) at 4 °C overnight in 1 × PBS, 5% (w/v), non-fat dried milk, and 0.1% Tween-20. For the cytosolic fraction, Western blots were also explored with antibody against β-actin protein (1:500, Santa Cruz Biotechnology, Dallas, TX, USA). The same methods were used for nuclear fraction with lamin A/C (1:500, Sigma-Aldrich Corp., Milan, Italy) [45, 54]. Signals were examined with an enhanced chemiluminescence (ECL) detection system reagent, according to the manufacturer’s instructions (Thermo, Monza, Italy). The relative expression of the protein bands was quantified by densitometry with BIORAD ChemiDocTM XRS^+^ software [55–59].

### Immunohistochemical Localization of TH, Dopamine Transporter (DAT), α-Synuclein, GFAP, and Iba-1

The immunohistochemical techniques used have been previously described [52, 58, 60]. Slices were incubated overnight with one of the following primary antibodies (specific for each whether polyclonal or monoclonal): anti-TH (Millipore, 1:500 in PBS, v/v), anti-DAT (Santa Cruz Biotechnology, 1:300 in PBS, v/v), anti-α-syn (Santa Cruz Biotechnology, 1:50 in PBS, v/v), anti-Iba-1 (Santa Cruz Biotechnology, 1:300 in PBS, v/v), and anti-GFAP (Santa Cruz Biotechnology; 1:200 in PBS, v/v). Immunohistochemical images were collected using Leica DM6 (Milan, Italy) associated with an Imaging system (LasX Navigator, Milan, Italy). The digital images were opened in ImageJ, followed by IHC profiler plug-in. All immunohistochemical analyses were carried out by two observers blinded to the treatment [29, 54, 61–63].

### Immunofluorescence Co-localization of TH/α-syn

Sections were incubated with the following primary antibodies: polyclonal anti-TH (1:250; Merck-Millipore) and monoclonal anti-α-syn (1:50; Santa Cruz Biotechnology) as previously described [51]. Sections were washed with PBS and were incubated with secondary antibody TEXAS RED-conjugated anti-rabbit Alexa Fluor-594 antibody (1:1000 in PBS, v/v Molecular Probes, UK) and with FITC-conjugated anti-mouse Alexa Fluor-488 antibody (1:2000 v/v Molecular Probes, UK) for 1 h at 37 °C. Sections were rinsed and stained for nuclear signal with 4′,6′-diamidino-2-phenylindole (DAPI; Hoechst, Frankfurt; Germany) 2 μg/ml in PBS. Sections were observed and photographed at × 100 magnification using a Leica DM2000 microscope.

### Tunel Staining

TUNEL staining protocol was according to a Roche protocol as previously described [45, 64–66]. Tunel staining was also incubated with anti-TH (1:250; Merck-Millipore) and FITC-conjugated anti-mouse Alexa Fluor-488 antibody (1:2000 v/v Molecular Probes, UK) for 1 h at 37 °C and then observed with Leica DM6 (Milan, Italy) associated with an Imaging system (LasX Navigator, Milan, Italy).

### Cytokine Measurement

TNF-α, IL-1β, and IL-6 levels were measured as previously described using a commercially available enzyme-linked immunosorbent assay (ELISA) (R&D Systems, Minneapolis, MN, USA) kits according to the manufacturer’s instructions [67].

### Myeloperoxidase and Malondialdehyde Measurement

MPO activity, an index of neutrophilic granulocyte infiltration, was evaluated as previously described and expressed as U/mg of tissue [52]. Lipid peroxidation were assessed with malonaldehyde as previously described and expressed as nmol/mg of proteins [68].

### Oxidative Stress and Antioxidant Defense

SOD, CAT, GPX, and GPx in the brain tissues were investigated as previously described [69, 70]. ROS content was measured using commercial kits according to manufacturer guidelines [71].

### Materials

Unless otherwise stated, all compounds were obtained from Sigma-Aldrich.

### Statistical Evaluation

In this study, the data are expressed as the average ± SEM and represent at least 3 experiments carried out in different days. For in vivo studies, N represents the number of animals used. The number of animals used for in vivo studies was carried out by G*Power 3.1 software (Die Heinrich-Heine-Universität Düsseldorf, Düsseldorf, Germany). Data were analyzed by an experienced histopathologist, and all the studies were performed without knowledge of the treatments. The results were analyzed by one-way ANOVA followed by a Bonferroni post hoc test for multiple comparisons. A *p* value less than 0.05 was considered significant.

## Results

### Açai Supplementation Reduces Both Motor and Non-motor Deficits

**Fig. 1:**
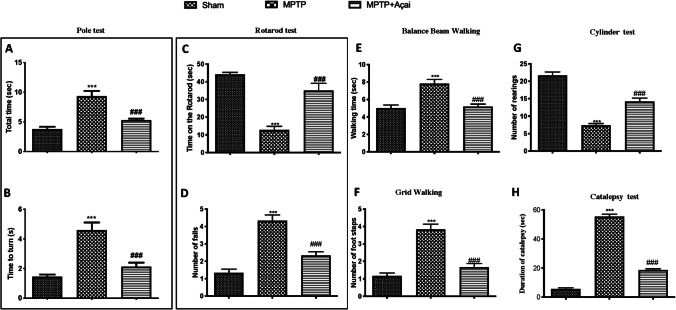
Açai supplementation reduces motor deficits. Total time (A) and time to turn (B) on pole test; time (C) and number of falls (D) on rotarod test; balance beam walking (E); number of foot on grid walking (F); cylinder test (G) and catalepsy test (H). See manuscript for further details. Values are means ± SEM of 6 mice for all group. ***p < 0.001 vs. sham; ###*p* < 0.001 vs. MPTP

**Fig. 2:**
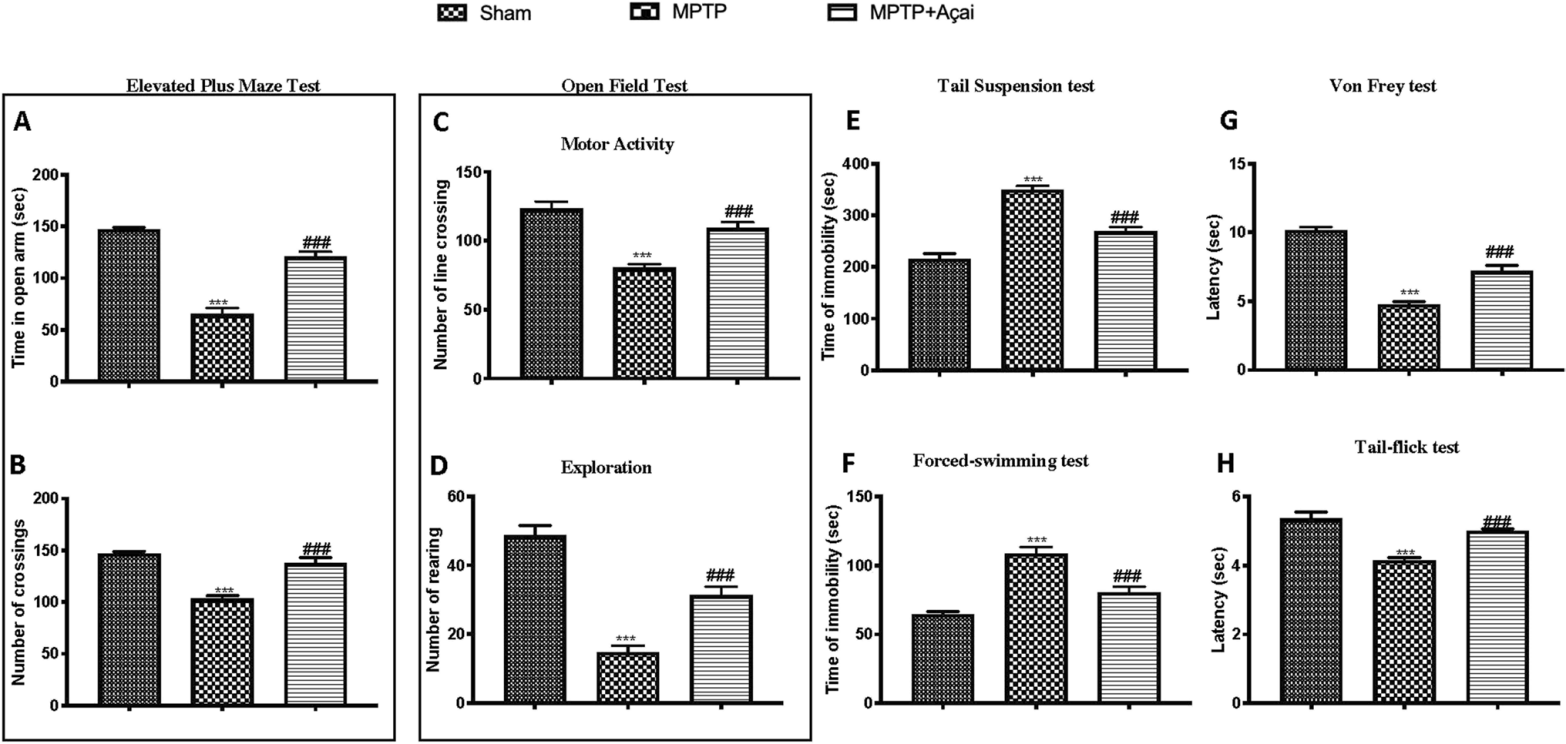
Açai supplementation reduces non motor deficits. Time in open arm (A) and number of crossing (B) during elevated plus maze test; number of line crossing (C) and number of rearing (D); tail suspension test (E); forced swimming test (F); latency during von Frey test (G) and latency during tail flick test (H). See manuscript for further details. Values are means ± SEM of 6 mice for all group. ***p < 0.001 vs. sham; ###*p* < 0.001 vs. MPTP

The most known symptoms that unfortunately afflict people with Parkinson’s are represented by motor alterations [72]. For this reason, we investigated by different behavioral test such as pole test (Fig. 1A and B), rotarod test (Fig. 1C and D), balance beam walking (Fig. 1E), grid walking (Fig. 1F), cylinder test (Fig. 1G), and catalepsy test (Fig. 1H) motor alteration MPTP-induced. Animals subjected to MPTP induction showed significantly motor alteration such as an increasing in the time spent on pole to as well as an increase in time spent on the rotarod apparatus and an increase in the time spent to reach the goal or to explore the space. After the daily oral administration with Açai we registered a significantly decrease in this alteration and an almost return to the physiological conditions of the animal. PD can also be considered a neuropsychiatric disorder [73]. Several neuropsychiatric symptoms are in fact related to emotional and cognitive problems [74]. Also, in this case, we investigated behavioral alteration with a series of tests useful to investigated anxiety, depression and pain. In particular, we used elevated plus maze test (Fig. 2A and B), open field test (Fig. 2C and D), tail suspension test (Fig. 2E), forced-swimming test (Fig. 2F), Von Frey test (Fig. 2G), and tail-flick test (Fig. 2H). As supposed, we found a significantly mood alterations after MPTP induction with an increase in anxiety and depression state and a reduction in nociceptive stimuli. Açai administration was able to reduce behavioral alterations restoring also nociceptive sensitivity.

### Açai Berry Limits Histological Alteration MPTP-Induced

**Fig. 3:**
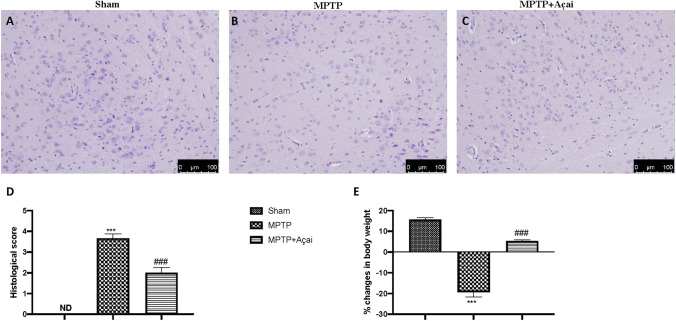
Açai berry limits histological alteration MPTP-induced. Brain section stained with H/E of Sham (A), MPTP (B), and MPTP + Açai (C); histological score (D); percentual in body weight changes (E). Scale bar 100 μm represents 20 × magnification. See manuscript for further details. Values are means ± SEM of 6 mice for all group. ***p < 0.001 vs. sham; ###*p* < 0.001 vs. MPTP

At the end of the experiment, brain samples were collected and stained for hematoxylin/eosin. Section of brain from the control group showing normal parenchymal and neurons (Fig. 3A and see relative histological score in Fig. 3D). Brain slices from MPTP group significantly showing alteration in brain tissue and a reduction in neuronal number (Fig. 3B and see relative histological score in Fig. 3D). Açai daily administration showing a marked reduction of degeneration and an increased number of SNpc neurons (Fig. 3C and see relative histological injury score in Fig. 3D). Additionally, we evaluated the decrease in body weight MPTP-induced. As shown in Fig. 3E, we observed a significantly reduction in body weight loss after 7 days of Açai administration.

### Açai Supplementation Restores TH and DAT Loss MPTP-Induced

**Fig. 4:**
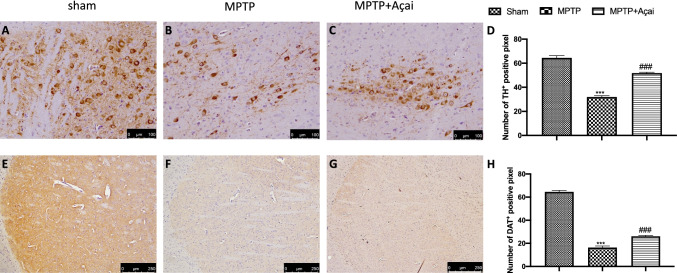
Açai supplementation restores TH and DAT loss MPTP-induced. Immunohistochemical localization of TH and DAT in brain section of Sham (A and E), MPTP (B and F) and MPTP + Açai (C and G); quantification of positive pixel of TH^+^ (D) and DAT^+^ (H). See manuscript for further details. Scale bar 100 μm represents 20 × magnification. Scale bar 250 μm represents 10 × magnification. Values are means ± SEM of 6 mice for all group. ***p < 0.001 vs. sham; ###*p* < 0.001 vs. MPTP

We assessed the degree of midbrain neuronal cell degeneration in terms of loss of TH^+^ in the substantia nigra and modification of DAT levels in the striatum because its well know that TH activity and DA levels are lowered in PD brain [51]. When MPTP-injected mice (Fig. 4B and F, see respectively densitometric analysis in Fig. 4D and H) were compared to sham mice (Fig. 4A and E, see respectively densitometric analysis in Fig. 4D and H), immunohistochemical examination revealed a clear decrease in terms of TH and DAT expression. Açai administration at the dose of 500 mg/kg for 7 days considerably restored TH and DAT levels (Fig. 4C and G, see respectively densitometric analysis in Fig. 4D and H).

### Açai Berry Reduce α-Syn Aggregation

**Fig. 5:**
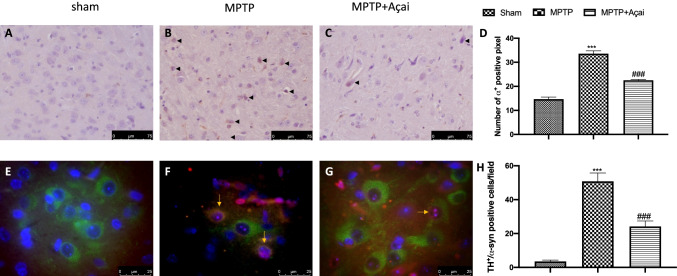
Açai berry reduce α-syn aggregation. Immunohistochemical localization of α-syn in brain section of Sham (A), MPTP (B), and MPTP + Açai (C); quantification of positive pixel of α-syn^+^ (D). Immunofluorescence co-localization on TH/α-syn in brain section of Sham (E), MPTP (F), and MPTP + Açai (G); number of positive cells/field (H). Yellow arrow indicates the expression of both markers. See manuscript for further details. Scale bar 75 μm represents 40 × magnification. Scale bar 25 μm represents 100 × magnification. Values are means ± SEM of 6 mice for all group. ***p < 0.001 vs. sham; ###*p* < 0.001 vs. MPTP

The Lewy body contains a lot of misfolded α-syn [75]. In comparison to sham animals (Fig. 5A and densitometric analysis in Fig. 5D), MPTP injection resulted in a large increase in α-syn accumulation (Fig. 5B and densitometric analysis in Fig. 5D). On the other hand, Açai administration was able to reduce the accumulation of misfolded α-syn (Fig. 5C and densitometric analysis in Fig. 5D). To better appreciate misfolded α-syn aggregation in dopaminergic neurons, we made immunofluorescence co-localization. We did not find any positive co-localization in sham animals (Fig. 5E and densitometric analysis in Fig. 5H), whereas MPTP injection resulted in a significantly α-syn accumulation in dopaminergic neurons (Fig. 5F and densitometric analysis in Fig. 5H). Açai administration significantly reduced the accumulation α-syn in dopaminergic neurons (Fig. 5G and densitometric analysis in Fig. 5H).

### Açai Supplementation Counteracts Astrogliosis and Microgliosis

**Fig. 6:**
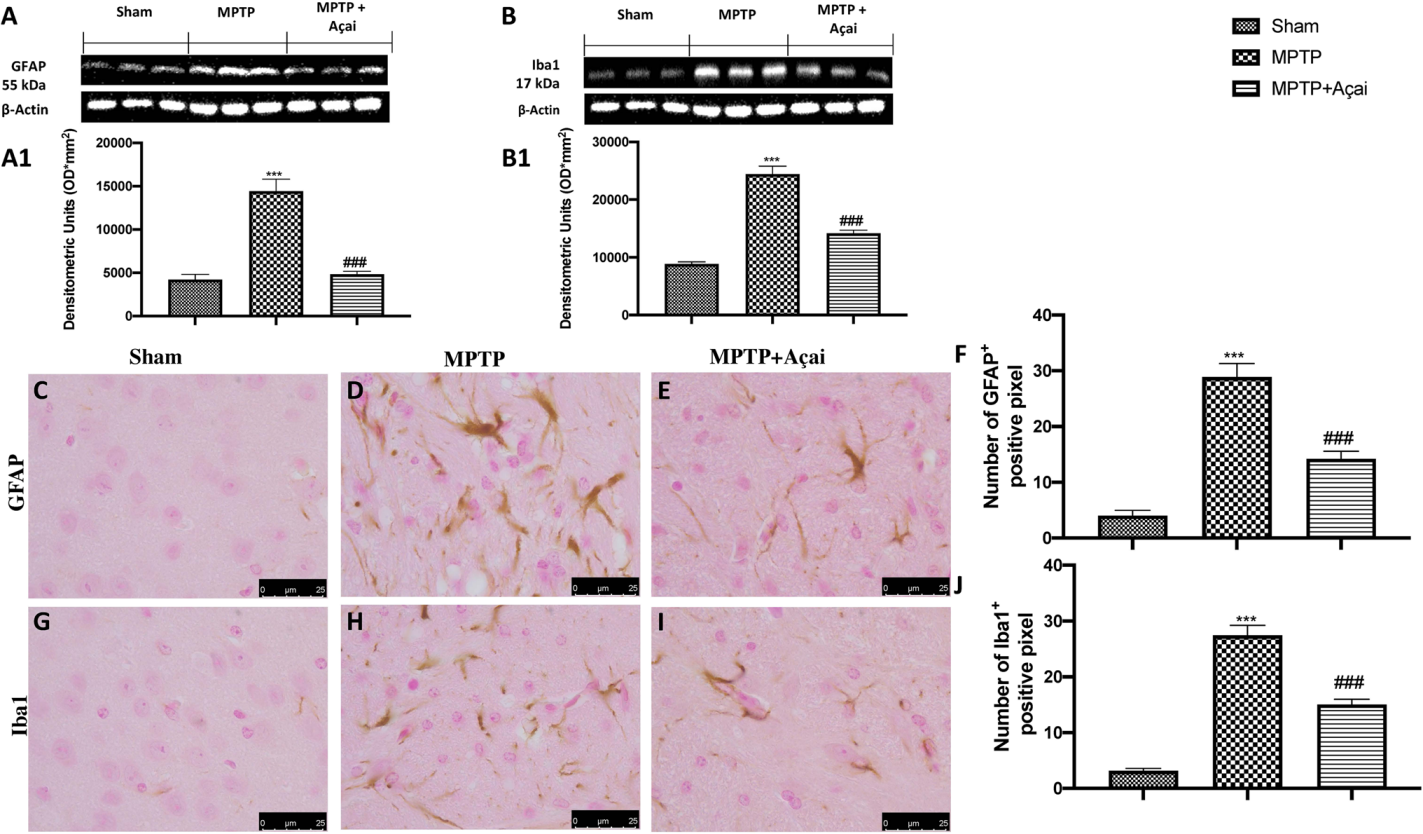
Açai supplementation counteract astrogliosis and microgliosis. Western blots and relative densitometric analysis of GFAP (A and A1) and Iba-1 (B and B1). Immunohistochemical localization of GFAP and Iba-1 in brain section of Sham (C and G), MPTP (D and H), and MPTP + Açai (E and I); quantification of positive pixel of GFAP^+^ (F) and Iba-1^+^ (J). See manuscript for further details. Scale bar 25 μm represents 100 × magnification. Values are means ± SEM of 6 mice for all group. ***p < 0.001 vs. sham; ###*p* < 0.001 vs. MPTP

While glia and astrocytes are required for maintaining homeostasis in the healthy brain, their malfunction contributes to neurodegeneration in a variety of illnesses, including PD. By western blots and immunohistochemical staining we investigates the expression of GFAP and Iba-1, well know markers of astrocytosyis and microgliosys. We notice that after MPTP induction, there were a significant increase in both GFAP (see Fig. 6A and relative densitometric analysis in Fig. 6A1 for western blot and Fig. 6D and relative densitometric analysis in 6F for immunohistochemical) and Iba-1 (see Fig. 6B and relative densitometric analysis in Fig. 6B1 for western blot and Fig. 6H and relative densitometric analysis in Fig. 6J for immunohistochemical) expressions compared to sham animals (see Fig. 6A and relative densitometric analysis in Fig. 6A1 for western blot and Fig. 6G and relative densitometric analysis in Fig. 6F for immunohistochemical of GFAP; Fig. 6B and relative densitometric analysis in Fig. 6B1 for western blot and Fig. 6G and relative densitometric analysis in Fig. 6J for immunohistochemical of Iba-1). Daily administration of Açai at the dose of 500 mg/kg significantly reduce both expressions (see Fig. 6A and relative densitometric analysis in Fig. 6A1 for western blot and Fig. 6E and relative densitometric analysis in Fig. 6F for immunohistochemical of GFAP; Fig. 6B and relative densitometric analysis in Fig. 6B1 for western blot and Fig. 6I and relative densitometric analysis in Fig. 6J for immunohistochemical of Iba-1).

### Açai Berry Reduces Proinflammatory Cytokine Release, Neutrophilic Infilitration, and Lipid Peroxidation

**Fig. 7:**
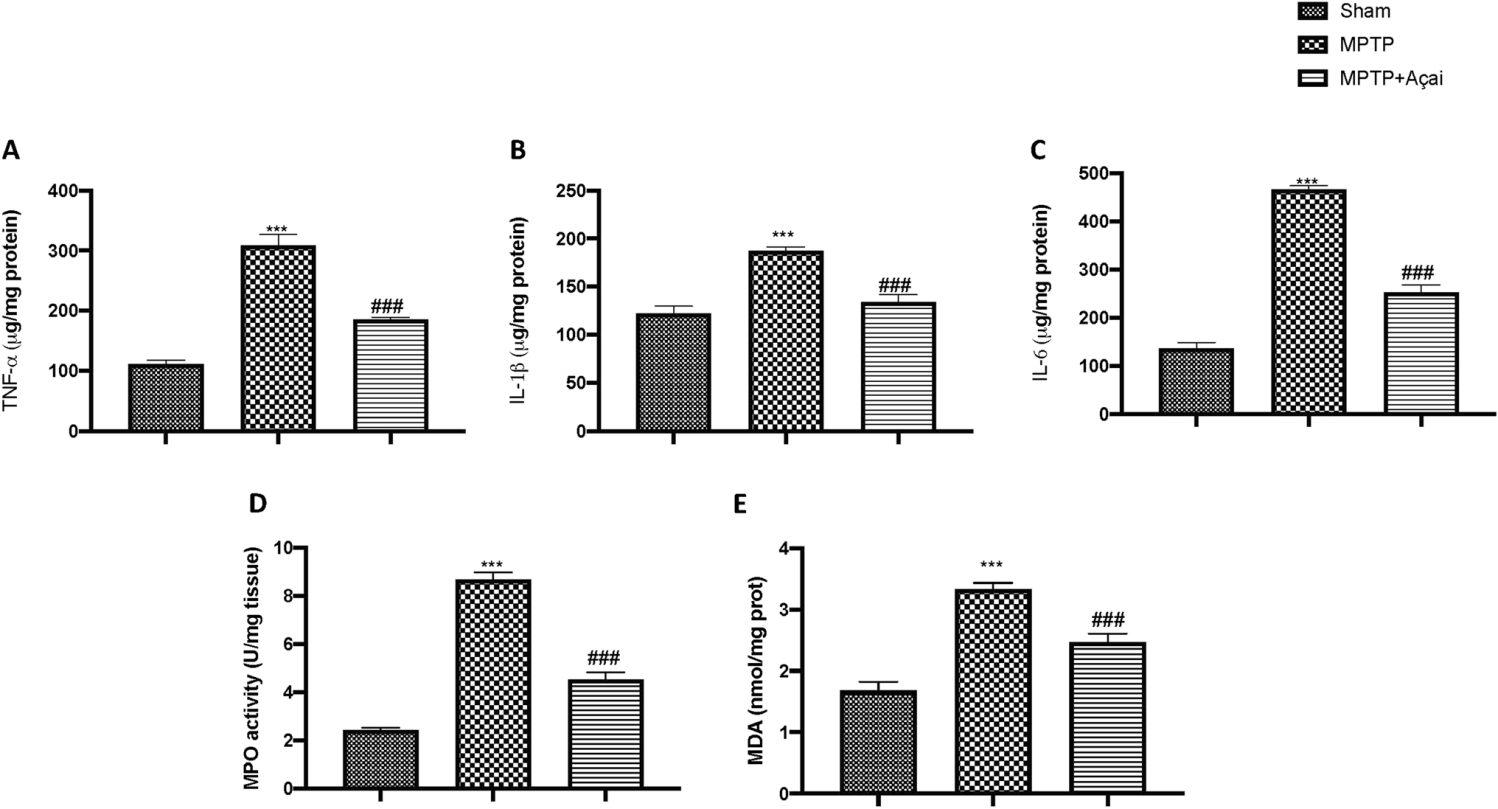
Açai berry reduce proinflammatory cytokine release, neutrophilic infilitration, and lipid peroxidation. ELISA quantification for TNF-α (A), IL-1β (B) and IL-6 (C) MPO quantification (D), and lipid peroxidation (E). See manuscript for further details. Values are means ± SEM of 6 mice for all group. ***p < 0.001 vs. sham; ###*p* < 0.001 vs. MPTP

MPTP triggers an inflammatory response that aids in the progression of neurodegeneration. The proinflammatory cytokines TNF-α, IL-1β, and IL-6 are produced by astrocytes and glia [76]. By ELISA kit, we investigated brain release of proinflammartory cytokines and we found a significantly increase in TNF-α (Fig. 7A), IL-1β (Fig. 7B), and IL-6 (Fig. 7C) after MPTP induction compared to sham group. As supposed, we found a significantly decrease after Daily administration of Açai. MPO and MDA levels in brain tissue have been found to be elevated in numerous neurodegenerative diseases [77, 78]. In accordance with the bibliography, we found a significantly increase in MPO and MDA levels after MPTP induction (Fig. 7D) compared to control group. On the other hand, Açai considerably decreases both.

### Açai Supplementation Improves Antioxidant Defense

**Fig. 8:**
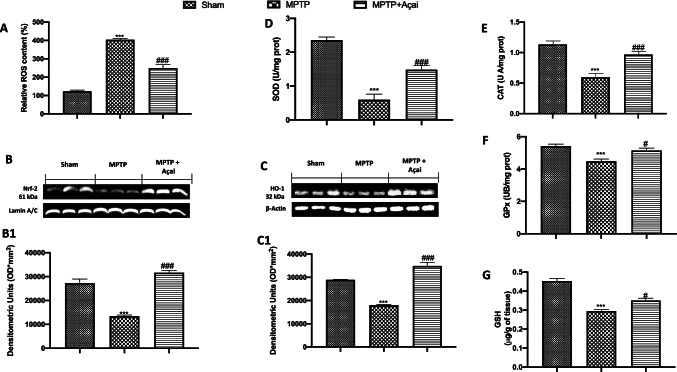
Açai supplementation improve antioxidant defense. ROS content (A); Western blots and relative densitometric analysis of Nrf-2 (B and B1) and HO-1 (C and C1); SOD (D); CAT(E); GPx (F) and GSH (G). See manuscript for further details. Values are means ± SEM of 6 mice for all group. ***p < 0.001 vs. sham; ###*p* < 0.001 vs. MPTP

In PD, oxidative stress plays a key role in the cascade that leads to dopamine cell destruction [79]. We investigated the oxidative stress by the analysis of ROS and antioxidant system, and we found that after MPTP induction, there were an increase in ROS production (Fig. 8A) and a decrease in Nrf-2 (Fig. 8B and relative densitometric analysis in Fig. 8B1) pathways as well as in HO-1 (Fig. 8C and relative densitometric analysis in Fig. 8C1), SOD (Fig. 8D), CAT (Fig. 8E), GPx (Fig. 8F), and GSH (Fig. 8G) compared to sham group. Açai administration 500 mg/kg significantly improve physiological antioxidant defense decreasing ROS production.

### Açai Berry Limits Dopaminergic Neuronal Death

**Fig. 9:**
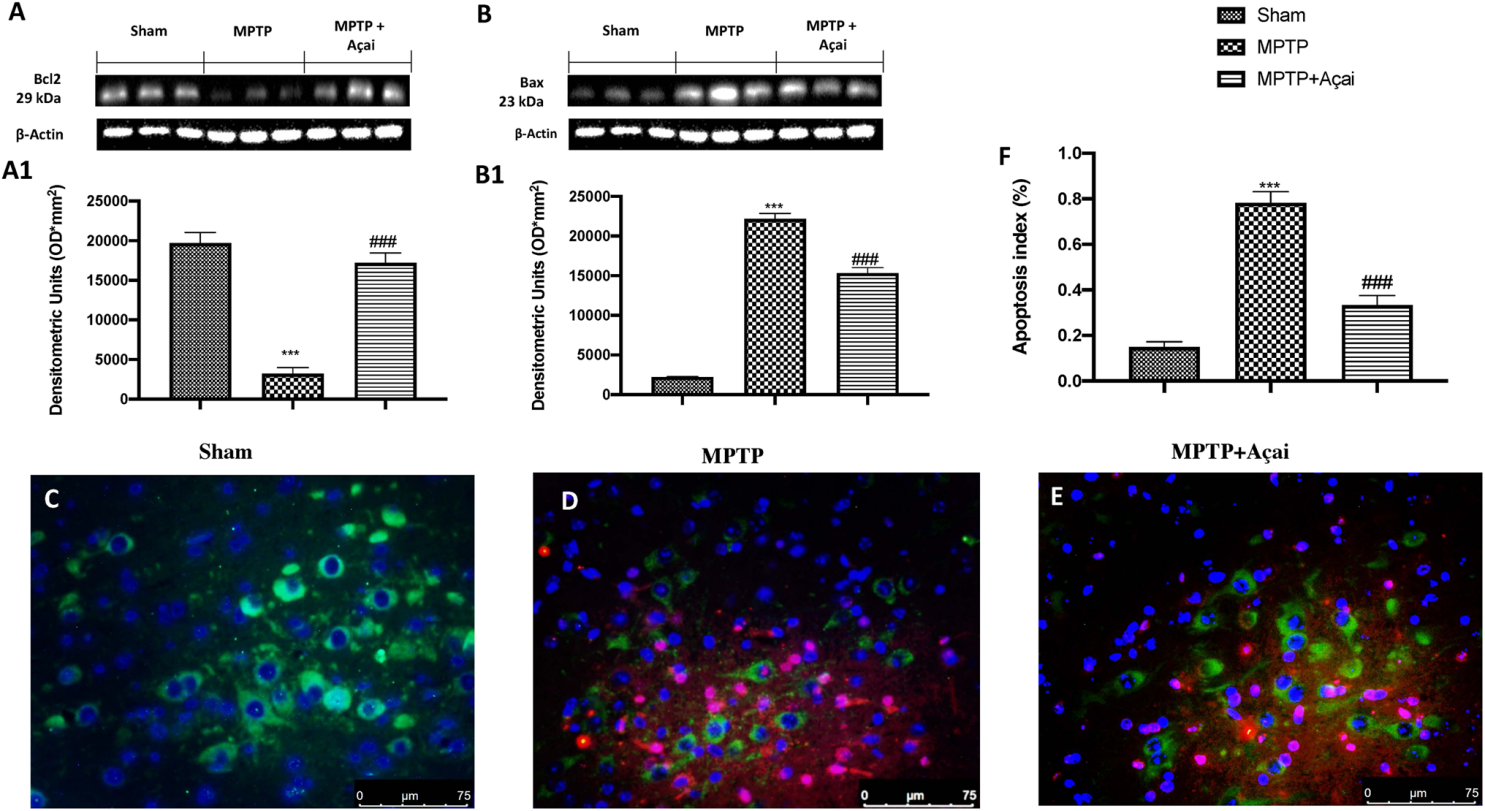
Açai Berry limits dopaminergic neuronal death. Western blots and relative densitometric analysis of Bcl-2 (A and A1) and Bax (B and B1); Immunofluorescence co-localization of TH/TUNEL in brain section of Sham (C), MPTP (D) and MPTP + Açai (E); apoptosis index expressed in percentual (F). See manuscript for further details. Values are means ± SEM of 6 mice for all group. ***p < 0.001 vs. sham; ###*p* < 0.001 vs. MPTP

By western blot and colocalization TH/TUNEL, we investigated neuronal death. We found that after Açai administration, there were a significant increase in Bcl-2 expression (Fig. 9A and relavive densitometric analysis in Fig. 9A1) as well as a considerably decrease in Bax expression (Fig. 9B and relavive densitometric analysis in Fig. 9B1) compared to MPTP group. To investigate in particular dopaminergic death, we analyzed TUNEL and TH expression and we found that MPTP significantly induce dopaminergic death (Fig. 9D and apoptotic index in Fig. 9F) compared to sham group (Fig. 9C and apoptotic index in Fig. 9F), whereas Açai at the dose of 500 mg/kg considerably reduces TH^+^ cell death.

## Discussions

PD is the most prevalent neurological movement disorder, with a global frequency of 0.1% and a prevalence of 3% in those over 65. After the loss of > 50% of dopaminergic (DAergic) neurons in the substantia nigra (SN) pars compacta and > 80% drop in DA levels in the striatum, motor symptoms such as bradykinesia, tremor, and stiffness appear. In addition, psychological comorbidities such as depression and anxiety are frequent in people with Parkinson’s disease, and they lead to considerable functional impairment as well as poor motor and social performance. This results in a lower quality of life and a greater strain on caregivers [80, 81]. Mood disorders are frequently misdiagnosed because their symptoms coincide with the cognitive and motor aspects of Parkinson's disease. As a result, early diagnosis and treatment for anxiety and depression are critical in the treatment of PD [82, 83]. The buildup of α-synuclein-rich protein aggregates, known as Lewy bodies, and a rise in the neuroinflammatory indicators of microgliosis and astrogliosis are the well know anatomopathological hallmarks of the illness [3]. Replacement of striatal DA is the focus of current pharmaceutical treatment. Levodopa crosses the blood–brain barrier and enters the presynaptic neurons via the DA transporter (DAT), where it is converted to DA and stored in vesicles. For decades, these medications, alone or in conjunction with pharmaceuticals that affect cholinergic modulation, have been of great help to most PD patients. However, while these methods alleviate motor symptoms, it is unclear if they aid in slowing the disease’s course [84]. Stopping the chain of events that leads to the development of PD is undoubtedly a major problem that necessitates a neuroprotective strategy to maintain the DAergic neurons that are still viable in the newly diagnosed patient alive and functional. To make progress in this area, a greater understanding of PD etiopathology is required, followed by the identification of molecular targets that might support the development of a neuroprotective medication in the clinic. Although there is no single cause of Parkinson’s disease, evidence from sporadic and familial cases, as well as chemical and genetic animal models, clearly shows that oxidative stress plays a key role in the illness’s onset and development. As a result, pharmaceutical intervention, whether or not to alleviate or counteract excessive ROS generation, might become a novel neuroprotective technique [2].

There is now a variety of early research suggesting that some foods may slow the course of Parkinson’s disease. These findings are not surprising, given that nutrients influence mitochondrial energy function and offer important antioxidant capabilities that reduce oxidative phosphorylation's free radical byproducts. Increased oxidative stress from a poor diet may compromise the antioxidant defense system. A well-balanced diet rich in a range of nutrients, such as several servings of vegetables and fruits, moderate doses of omega-3 fatty acids, tea, coffee, and wine, on the other hand, may give neuroprotection [11, 85]. The new food, generally known as “Açai,” is a berry native to South America that belongs to the Euterpe genus of tropical palm plants. Scientists have been studying *Euterpe oleracea* because of its high antioxidant content when compared to other fruits and berries. Açai pulp composition research also revealed that it includes several physiologically active phytochemicals. Açai berries have been shown to have neuroprotective qualities in a number of studies [24]. Many of these diseases are multifactorial, resulting from a combination of aging, genetic disorders, and exposure to one or more environmental factors, which cause oxidative stress, chronic neuroinflammation, excitotoxicity, mitochondrial dysfunction, and irregular protein accumulation in brain tissues, among other cellular etiologies. Experiments showed that Açai berry extracts provide neuroprotection by exhibiting antioxidant and anti-inflammatory properties, suppressing harmful protein aggregation, and restoring calcium homeostasis and mitochondrial function, among other things. Açai fruit also has antidepressant and anticonvulsant properties, which might be useful to persons with these neurodisorders [86–93]. With this aim in our mind, we used a consolidated murine model of PD to investigates beneficial effects of Açai supplementation in behavioural disorders as well as against astrogliosis and microgliosis, oxidative stress and apoptosis.

In our study using different behavioral tests, we found that Açai supplementation was in grade to reduces both motor and non motor deficits limiting axiety and depression state as well as tremor, bradikynesia and stiffness. Additionally, we found that that Açai berry supplementation at the dose of 500 mg/kg administred daily limits histological alteration in the substantia nigra MPTP-induced restoring TH and DAT expression as well as was able to reduce α-syn aggregation.

In accordance with the bibliography, we found that Açai berry supplementation was able to counteract astrogliosis and microgliosis as well as proinflammatory cytokine release, neutrophilic infilitration and lipid peroxidation. These beneficial effects are probably due to effects that Açai berry showing on physiological anti oxidant defence. We found that Açai Berry supplementation at the dose of 500 mg/kg administred daily significantly improve Nrf-2 expression as well as HO-1, SOD, CAT, GPx, and GSH reducing oxidative stress general state.

The improvement of anti oxidant defence was also reflected in the reduction of neuronal death with particular attention on dopaminergic death. In conclusion with our work, we confirmed that diet is the best medicine in several disorders, including neurodegenerative disease and in particular we demonstrated for the first time that Açai berry supplementation at the dose of 500 mg/kg was useful to counteract the neuroinflammatory and oxidative events characteristic of the PD, limiting neuronal death and improving physiological antioxidant defense.

## Data Availability

The datasets generated and/or analyzed for the present study are available from the corresponding author on reasonable request.

## References

[1] Warner TT, Schapira AH (2003) Genetic and environmental factors in the cause of Parkinson's disease. Ann Neurol 53 Suppl 3:S16–23; discussion S23–15 10.1002/ana.10487 10.1002/ana.10487 12666095

[2] Rodriguez-Oroz MC, Jahanshahi M, Krack P, Litvan I, Macias R, Bezard E, Obeso JA (2009). Initial clinical manifestations of Parkinson's disease: features and pathophysiological mechanisms. Lancet Neurol.

[3] Moore DJ, West AB, Dawson VL, Dawson TM (2005). Molecular pathophysiology of Parkinson's disease. Annu Rev Neurosci.

[4] Smith PF (2008). Inflammation in Parkinson's disease: an update. Curr Opin Investig Drugs.

[5] Gureev AP, Popov VN (2019). Nrf2/ARE pathway as a therapeutic target for the treatment of parkinson diseases. Neurochem Res.

[6] Lee JM, Calkins MJ, Chan K, Kan YW, Johnson JA (2003). Identification of the NF-E2-related factor-2-dependent genes conferring protection against oxidative stress in primary cortical astrocytes using oligonucleotide microarray analysis. J Biol Chem.

[7] Lee JM, Shih AY, Murphy TH, Johnson JA (2003). NF-E2-related factor-2 mediates neuroprotection against mitochondrial complex I inhibitors and increased concentrations of intracellular calcium in primary cortical neurons. J Biol Chem.

[8] Thimmulappa RK, Mai KH, Srisuma S, Kensler TW, Yamamoto M, Biswal S (2002). Identification of Nrf2-regulated genes induced by the chemopreventive agent sulforaphane by oligonucleotide microarray. Cancer Res.

[9] Ramsey CP, Glass CA, Montgomery MB, Lindl KA, Ritson GP, Chia LA, Hamilton RL, Chu CT, Jordan-Sciutto KL (2007). Expression of Nrf2 in neurodegenerative diseases. J Neuropathol Exp Neurol.

[10] Cuadrado A, Moreno-Murciano P, Pedraza-Chaverri J (2009). The transcription factor Nrf2 as a new therapeutic target in Parkinson's disease. Expert Opin Ther Targets.

[11] Seidl SE, Santiago JA, Bilyk H, Potashkin JA (2014). The emerging role of nutrition in Parkinson's disease. Front Aging Neurosci.

[12] Liu RH (2003). Health benefits of fruit and vegetables are from additive and synergistic combinations of phytochemicals. Am J Clin Nutr.

[13] Gao X, Chen H, Fung TT, Logroscino G, Schwarzschild MA, Hu FB, Ascherio A (2007). Prospective study of dietary pattern and risk of Parkinson disease. Am J Clin Nutr.

[14] Okubo H, Miyake Y, Sasaki S, Murakami K, Tanaka K, Fukushima W, Kiyohara C, Tsuboi Y, Yamada T, Oeda T, Shimada H, Kawamura N, Sakae N, Fukuyama H, Hirota Y, Nagai M, Fukuoka Kinki Parkinson's Disease Study G (2012) Dietary patterns and risk of Parkinson's disease: a case-control study in Japan. Eur J Neurol 19(5):681–68810.1111/j.1468-1331.2011.03600.x 10.1111/j.1468-1331.2010.03088.x 20491891

[15] Maciel-Silva FW, Buller LS, MBB Gonçalves ML, Rostagno MA, Forster-Carneiro T (2021) Sustainable development in the Legal Amazon: energy recovery from açaí seeds. Biofuels Bioprod Biorefin 15(4):1174–1189

[16] Melo PS, Massarioli AP, Lazarini JG, Soares JC, Franchin M, Rosalen PL, Alencar SM (2020). Simulated gastrointestinal digestion of Brazilian acai seeds affects the content of flavan-3-ol derivatives, and their antioxidant and anti-inflammatory activities. Heliyon.

[17] Melo PS, Selani MM, Gonçalves RH, de Oliveira PJ, Massarioli AP, de Alencar SM (2021). Açaí seeds: An unexplored agro-industrial residue as a potential source of lipids, fibers, and antioxidant phenolic compounds. Ind Crops Prod.

[18] Rodrigues RB, Lichtenthaler R, Zimmermann BF, Papagiannopoulos M, Fabricius H, Marx F, Maia JG, Almeida O (2006) Total oxidant scavenging capacity of Euterpe oleracea Mart. (acai) seeds and identification of their polyphenolic compounds. J Agric Food Chem 54(12):4162–4167 10.1021/jf058169p 10.1021/jf058169p 16756342

[19] de Moura RS, Pires KM, Santos Ferreira T, Lopes AA, Nesi RT, Resende AC, Sousa PJ, da Silva AJ, Porto LC, Valenca SS (2011). Addition of acai (Euterpe oleracea) to cigarettes has a protective effect against emphysema in mice. Food Chem Toxicol.

[20] Lee JY, Kim N, Choi YJ, Nam RH, Lee S, Ham MH, Suh JH, Choi YJ, Lee HS, Lee DH (2016). Anti-inflammatory and anti-tumorigenic effects of Acai berry in Helicobacter felis-infected mice. J Cancer Prev.

[21] Moura RS, Ferreira TS, Lopes AA, Pires KM, Nesi RT, Resende AC, Souza PJ, Silva AJ, Borges RM, Porto LC, Valenca SS (2012) Effects of Euterpe oleracea Mart. (ACAI) extract in acute lung inflammation induced by cigarette smoke in the mouse. Phytomedicine 19(3–4):262–269 10.1016/j.phymed.2011.11.004 10.1016/j.phymed.2011.11.004 22138278

[22] Poulose SM, Fisher DR, Larson J, Bielinski DF, Rimando AM, Carey AN, Schauss AG, Shukitt-Hale B (2012) Anthocyanin-rich acai (Euterpe oleracea Mart.) fruit pulp fractions attenuate inflammatory stress signaling in mouse brain BV-2 microglial cells. J Agric Food Chem 60(4):1084–1093 10.1021/jf203989k 10.1021/jf203989k 22224493

[23] Santos IB, de Bem GF, da Costa CA, de Carvalho L, de Medeiros AF, Silva DLB, Romao MH, de Andrade SR, Ognibene DT, de Moura RS, Resende AC (2020). Acai seed extract prevents the renin-angiotensin system activation, oxidative stress and inflammation in white adipose tissue of high-fat diet-fed mice. Nutr Res.

[24] Al Nasser MN, Mellor IR (2022) Neuroprotective activities of acai berries (Euterpe sp.): a review. J Herbmed Pharmacol 11(2):166–181

[25] Kang J, Li Z, Wu T, Jensen GS, Schauss AG, Wu X (2010) Anti-oxidant capacities of flavonoid compounds isolated from acai pulp (Euterpe oleracea Mart.). Food Chem 122(3):610–617

[26] Siracusa R, Paterniti I, Cordaro M, Crupi R, Bruschetta G, Campolo M, Cuzzocrea S, Esposito E (2017). Neuroprotective Effects of Temsirolimus in Animal Models of Parkinson's Disease. Mol Neurobiol.

[27] Sedelis M, Schwarting RK, Huston JP (2001). Behavioral phenotyping of the MPTP mouse model of Parkinson's disease. Behav Brain Res.

[28] Fleming SM, Mulligan CK, Richter F, Mortazavi F, Lemesre V, Frias C, Zhu C, Stewart A, Gozes I, Morimoto B, Chesselet MF (2011). A pilot trial of the microtubule-interacting peptide (NAP) in mice overexpressing alpha-synuclein shows improvement in motor function and reduction of alpha-synuclein inclusions. Mol Cell Neurosci.

[29] Siracusa R, Paterniti I, Cordaro M, Crupi R, Bruschetta G, Campolo M, Cuzzocrea S, Esposito E (2018). Neuroprotective effects of temsirolimus in animal models of Parkinson's disease. Mol Neurobiol.

[30] Hou X, Yuan Y, Sheng Y, Yuan B, Wang Y, Zheng J, Liu CF, Zhang X, Hu LF (2017). GYY4137, an H2S slow-releasing donor, prevents nitrative stress and alpha-synuclein nitration in an MPTP mouse model of Parkinson's disease. Front Pharmacol.

[31] Bhurtel S, Katila N, Srivastav S, Neupane S, Choi DY (2019). Mechanistic comparison between MPTP and rotenone neurotoxicity in mice. Neurotoxicology.

[32] Araki T, Kumagai T, Tanaka K, Matsubara M, Kato H, Itoyama Y, Imai Y (2001). Neuroprotective effect of riluzole in MPTP-treated mice. Brain Res.

[33] Paterniti I, Campolo M, Siracusa R, Cordaro M, Di Paola R, Calabrese V, Navarra M, Cuzzocrea S, Esposito E (2017). Liver X receptors activation, through TO901317 binding, reduces neuroinflammation in Parkinson's disease. PLoS ONE.

[34] Bortolato M, Godar SC, Davarian S, Chen K, Shih JC (2009). Behavioral disinhibition and reduced anxiety-like behaviors in monoamine oxidase B-deficient mice. Neuropsychopharmacology.

[35] Pellow S, Chopin P, File SE, Briley M (1985). Validation of open:closed arm entries in an elevated plus-maze as a measure of anxiety in the rat. J Neurosci Methods.

[36] Papale A, d'Isa R, Menna E, Cerovic M, Solari N, Hardingham N, Cambiaghi M, Cursi M, Barbacid M, Leocani L, Fasano S, Matteoli M, Brambilla R (2017). Severe intellectual disability and enhanced gamma-aminobutyric acidergic synaptogenesis in a novel model of rare RASopathies. Biol Psychiatry.

[37] Prut L, Belzung C (2003). The open field as a paradigm to measure the effects of drugs on anxiety-like behaviors: a review. Eur J Pharmacol.

[38] Crupi R, Cambiaghi M, Spatz L, Hen R, Thorn M, Friedman E, Vita G, Battaglia F (2010). Reduced adult neurogenesis and altered emotional behaviors in autoimmune-prone B-cell activating factor transgenic mice. Biol Psychiatry.

[39] Gorton LM, Vuckovic MG, Vertelkina N, Petzinger GM, Jakowec MW, Wood RI (2010). Exercise effects on motor and affective behavior and catecholamine neurochemistry in the MPTP-lesioned mouse. Behav Brain Res.

[40] Porsolt RD, Bertin A, Blavet N, Deniel M, Jalfre M (1979). Immobility induced by forced swimming in rats: effects of agents which modify central catecholamine and serotonin activity. Eur J Pharmacol.

[41] Crupi R, Mazzon E, Marino A, La Spada G, Bramanti P, Cuzzocrea S, Spina E (2010). Melatonin treatment mimics the antidepressant action in chronic corticosterone-treated mice. J Pineal Res.

[42] Abolarin PO, Nafiu AB, Oyewole AL, Amin A, Ogundele OM, Owoyele BV (2022). Selenium reduces nociceptive response in acute 1-methyl-4-phenyl-1, 2, 3, 6-tetrahydropyridine (MPTP)-induced neurotoxicity. IBRO Neurosci Rep.

[43] Siracusa R, Paterniti I, Impellizzeri D, Cordaro M, Crupi R, Navarra M, Cuzzocrea S, Esposito E (2015). The association of palmitoylethanolamide with luteolin decreases neuroinflammation and stimulates autophagy in Parkinson's disease model. CNS Neurol Disord Drug Targets.

[44] Esposito E, Impellizzeri D, Bruschetta G, Cordaro M, Siracusa R, Gugliandolo E, Crupi R, Cuzzocrea S (2016). A new co-micronized composite containing palmitoylethanolamide and polydatin shows superior oral efficacy compared to their association in a rat paw model of carrageenan-induced inflammation. Eur J Pharmacol.

[45] Gugliandolo E, D'Amico R, Cordaro M, Fusco R, Siracusa R, Crupi R, Impellizzeri D, Cuzzocrea S, Di Paola R (2018). Effect of PEA-OXA on neuropathic pain and functional recovery after sciatic nerve crush. J Neuroinflammation.

[46] Gugliandolo E, D'Amico R, Cordaro M, Fusco R, Siracusa R, Crupi R, Impellizzeri D, Cuzzocrea S, Di Paola R (2018). Neuroprotective effect of artesunate in experimental model of traumatic brain injury. Front Neurol.

[47] Impellizzeri D, Cordaro M, Bruschetta G, Crupi R, Pascali J, Alfonsi D, Marcolongo G, Cuzzocrea S (2016). 2-pentadecyl-2-oxazoline: identification in coffee, synthesis and activity in a rat model of carrageenan-induced hindpaw inflammation. Pharmacol Res.

[48] Cordaro M, Paterniti I, Siracusa R, Impellizzeri D, Esposito E, Cuzzocrea S (2016). KU0063794, a dual mTORC1 and mTORC2 inhibitor, reduces neural tissue damage and locomotor impairment after spinal cord injury in mice. Mol Neurobiol.

[49] Bertolino B, Crupi R, Impellizzeri D, Bruschetta G, Cordaro M, Siracusa R, Esposito E, Cuzzocrea S (2017). Beneficial effects of co-ultramicronized palmitoylethanolamide/luteolin in a mouse model of autism and in a case report of autism. CNS Neurosci Ther.

[50] Britti D, Crupi R, Impellizzeri D, Gugliandolo E, Fusco R, Schievano C, Morittu VM, Evangelista M, Di Paola R, Cuzzocrea S (2017). A novel composite formulation of palmitoylethanolamide and quercetin decreases inflammation and relieves pain in inflammatory and osteoarthritic pain models. BMC Vet Res.

[51] Cordaro M, Siracusa R, Crupi R, Impellizzeri D, Peritore AF, D'Amico R, Gugliandolo E, Di Paola R, Cuzzocrea S (2018). 2-Pentadecyl-2-oxazoline reduces neuroinflammatory environment in the MPTP model of Parkinson disease. Mol Neurobiol.

[52] Cordaro M, Siracusa R, Impellizzeri D, R DA, Peritore AF, Crupi R, Gugliandolo E, Fusco R, Di Paola R, Schievano C, Cuzzocrea S (2019) Safety and efficacy of a new micronized formulation of the ALIAmide palmitoylglucosamine in preclinical models of inflammation and osteoarthritis pain. Arthritis Res Ther 21(1):25410.1186/s13075-019-2048-y 10.1186/s13075-019-2048-y PMC6883534 31779692

[53] Crupi R, Impellizzeri D, Bruschetta G, Cordaro M, Paterniti I, Siracusa R, Cuzzocrea S, Esposito E (2016). Co-ultramicronized palmitoylethanolamide/luteolin promotes neuronal regeneration after spinal cord injury. Front Pharmacol.

[54] Fusco R, D'Amico R, Cordaro M, Gugliandolo E, Siracusa R, Peritore AF, Crupi R, Impellizzeri D, Cuzzocrea S, Di Paola R (2018). Absence of formyl peptide receptor 1 causes endometriotic lesion regression in a mouse model of surgically-induced endometriosis. Oncotarget.

[55] Fusco R, Siracusa R, Peritore AF, Gugliandolo E, Genovese T, D'Amico R, Cordaro M, Crupi R, Mandalari G, Impellizzeri D, Cuzzocrea S, Di Paola R (2020) The role of cashew (Anacardium occidentale L.) nuts on an experimental model of painful degenerative joint disease. Antioxidants (Basel) 9(6) 10.3390/antiox9060511 10.3390/antiox9060511 PMC7346149 32532064

[56] Siracusa R, Fusco R, Peritore AF, Cordaro M, D'Amico R, Genovese T, Gugliandolo E, Crupi R, Smeriglio A, Mandalari G, Cuzzocrea S, Di Paola R, Impellizzeri D (2020) The antioxidant and anti-inflammatory properties of Anacardium occidentale L. cashew nuts in a mouse model of colitis. Nutrients 12(3) 10.3390/nu12030834 10.3390/nu12030834 PMC7146548 32245085

[57] Di Paola R, Cordaro M, Crupi R, Siracusa R, Campolo M, Bruschetta G, Fusco R, Pugliatti P, Esposito E, Cuzzocrea S (2016). Protective effects of ultramicronized palmitoylethanolamide (PEA-um) in myocardial ischaemia and reperfusion injury in vivo. Shock.

[58] Di Paola R, Fusco R, Impellizzeri D, Cordaro M, Britti D, Morittu VM, Evangelista M, Cuzzocrea S (2016). Adelmidrol, in combination with hyaluronic acid, displays increased anti-inflammatory and analgesic effects against monosodium iodoacetate-induced osteoarthritis in rats. Arthritis Res Ther.

[59] Siracusa R, Impellizzeri D, Cordaro M, Crupi R, Esposito E, Petrosino S, Cuzzocrea S (2017). Anti-inflammatory and neuroprotective effects of Co-UltraPEALut in a mouse model of vascular dementia. Front Neurol.

[60] Cordaro M, Impellizzeri D, Gugliandolo E, Siracusa R, Crupi R, Esposito E, Cuzzocrea S (2016). Adelmidrol, a palmitoylethanolamide analogue, as a new pharmacological treatment for the management of inflammatory bowel disease. Mol Pharmacol.

[61] Peritore AF, Siracusa R, Fusco R, Gugliandolo E, D'Amico R, Cordaro M, Crupi R, Genovese T, Impellizzeri D, Cuzzocrea S, Di Paola R (2020) Ultramicronized palmitoylethanolamide and paracetamol, a new association to relieve hyperalgesia and pain in a sciatic nerve injury model in rat. Int J Mol Sci 21(10) 10.3390/ijms21103509 10.3390/ijms21103509 PMC7278943 32429243

[62] Impellizzeri D, Siracusa R, Cordaro M, Crupi R, Peritore AF, Gugliandolo E, D'Amico R, Petrosino S, Evangelista M, Di Paola R, Cuzzocrea S (2019). N-Palmitoylethanolamine-oxazoline (PEA-OXA): A new therapeutic strategy to reduce neuroinflammation, oxidative stress associated to vascular dementia in an experimental model of repeated bilateral common carotid arteries occlusion. Neurobiol Dis.

[63] Impellizzeri D, Siracusa R, Cordaro M, Peritore AF, Gugliandolo E, Mancuso G, Midiri A, Di Paola R, Cuzzocrea S (2018). Therapeutic potential of dinitrobenzene sulfonic acid (DNBS)-induced colitis in mice by targeting IL-1beta and IL-18. Biochem Pharmacol.

[64] Fusco R, Scuto M, Cordaro M, D'Amico R, Gugliandolo E, Siracusa R, Peritore AF, Crupi R, Impellizzeri D, Cuzzocrea S, Di Paola R (2019) N-palmitoylethanolamide-oxazoline protects against middle cerebral artery occlusion injury in diabetic rats by regulating the SIRT1 pathway. Int J Mol Sci 20(19) 10.3390/ijms20194845 10.3390/ijms20194845 PMC6801841 31569558

[65] D'Amico R, Siracusa R, Fusco R, Cordaro M, Genovese T, Peritore AF, Gugliandolo E, Crupi R, Impellizzeri D, Cuzzocrea S, Paola RD (2020) Protective effects of Colomast((R)), a new formulation of adelmidrol and sodium hyaluronate, in a mouse model of acute restraint stress. Int J Mol Sci 21(21) 10.3390/ijms21218136 10.3390/ijms21218136 PMC7662642 33143356

[66] D'Amico R, Fusco R, Cordaro M, Siracusa R, Peritore AF, Gugliandolo E, Crupi R, Scuto M, Cuzzocrea S, Di Paola R, Impellizzeri D (2020) Modulation of NLRP3 inflammasome through formyl peptide receptor 1 (Fpr-1) pathway as a new therapeutic target in bronchiolitis obliterans syndrome. Int J Mol Sci 21(6) 10.3390/ijms21062144 10.3390/ijms21062144 PMC7139667 32244997

[67] Cordaro M, Scuto M, Siracusa R, D'Amico R, Filippo Peritore A, Gugliandolo E, Fusco R, Crupi R, Impellizzeri D, Pozzebon M, Alfonsi D, Mattei N, Marcolongo G, Evangelista M, Cuzzocrea S, Di Paola R (2020). Effect of N-palmitoylethanolamine-oxazoline on comorbid neuropsychiatric disturbance associated with inflammatory bowel disease. FASEB J.

[68] Di Paola R, Crisafulli C, Mazzon E, Genovese T, Paterniti I, Bramanti P, Cuzzocrea S (2009). Effect of PD98059, a selective MAPK3/MAPK1 inhibitor, on acute lung injury in mice. Int J Immunopathol Pharmacol.

[69] Marklund S, Marklund G (1974). Involvement of the superoxide anion radical in the autoxidation of pyrogallol and a convenient assay for superoxide dismutase. Eur J Biochem.

[70] Rajasankar S, Manivasagam T, Surendran S (2009). Ashwagandha leaf extract: a potential agent in treating oxidative damage and physiological abnormalities seen in a mouse model of Parkinson's disease. Neurosci Lett.

[71] Fan Y, Maghimaa M, Chinnathambi A, Alharbi SA, Veeraraghavan VP, Mohan SK, Hussain S, Rengarajan T (2021). Tomentosin reduces behavior deficits and neuroinflammatory response in MPTP-induced Parkinson's disease in mice. J Environ Pathol Toxicol Oncol.

[72] Coelho M, Ferreira J, Rosa M, Sampaio C (2008). Treatment options for non-motor symptoms in late-stage Parkinson's disease. Expert Opin Pharmacother.

[73] Agid Y (2010). Parkinson's diseases: power of search. Rev Neurol (Paris).

[74] Castrioto A, Thobois S, Carnicella S, Maillet A, Krack P (2016). Emotional manifestations of PD: Neurobiological basis. Mov Disord.

[75] Campolo M, Casili G, Biundo F, Crupi R, Cordaro M, Cuzzocrea S, Esposito E (2017). The neuroprotective effect of dimethyl fumarate in an MPTP-mouse model of Parkinson's disease: involvement of reactive oxygen species/nuclear factor-kappaB/nuclear transcription factor related to NF-E2. Antioxid Redox Signal.

[76] Stojkovska I, Wagner BM, Morrison BE (2015). Parkinson's disease and enhanced inflammatory response. Exp Biol Med (Maywood).

[77] Gellhaar S, Sunnemark D, Eriksson H, Olson L, Galter D (2017). Myeloperoxidase-immunoreactive cells are significantly increased in brain areas affected by neurodegeneration in Parkinson's and Alzheimer's disease. Cell Tissue Res.

[78] Xiao YL, Fu JM, Dong Z, Yang JQ, Zeng FX, Zhu LX, He BC (2004). Neuroprotective mechanism of modafinil on Parkinson disease induced by 1-methyl-4-phenyl-1,2,3,6-tetrahydropyridine. Acta Pharmacol Sin.

[79] Dias V, Junn E, Mouradian MM (2013). The role of oxidative stress in Parkinson's disease. J Parkinsons Dis.

[80] Bach JP, Riedel O, Klotsche J, Spottke A, Dodel R, Wittchen HU (2012). Impact of complications and comorbidities on treatment costs and health-related quality of life of patients with Parkinson's disease. J Neurol Sci.

[81] Jones JD, Butterfield LC, Song W, Lafo J, Mangal P, Okun MS, Bowers D (2015). Anxiety and depression are better correlates of Parkinson's disease quality of life than apathy. J Neuropsychiatry Clin Neurosci.

[82] Ray S, Agarwal P (2020). Depression and anxiety in Parkinson disease. Clin Geriatr Med.

[83] Weintraub D, Moberg PJ, Duda JE, Katz IR, Stern MB (2004). Effect of psychiatric and other nonmotor symptoms on disability in Parkinson's disease. J Am Geriatr Soc.

[84] Jenner P (2007). Oxidative stress and Parkinson's disease. Handb Clin Neurol.

[85] Barichella M, Cereda E, Pezzoli G (2009). Major nutritional issues in the management of Parkinson's disease. Mov Disord.

[86] Chen WW, Zhang X, Huang WJ (2016). Role of neuroinflammation in neurodegenerative diseases (Review). Mol Med Rep.

[87] de Almeida Magalhaes TSS, de Oliveira Macedo PC, Converti A, Neves de Lima AA (2020) The use of Euterpe oleracea Mart. as a new perspective for disease treatment and prevention. Biomolecules 10 (6) 10.3390/biom10060813 10.3390/biom10060813 PMC7356995 32466439

[88] Denzer I, Munch G, Friedland K (2016). Modulation of mitochondrial dysfunction in neurodegenerative diseases via activation of nuclear factor erythroid-2-related factor 2 by food-derived compounds. Pharmacol Res.

[89] Kovacs GG (2016) Molecular pathological classification of neurodegenerative diseases: turning towards precision medicine. Int J Mol Sci 17(2) 10.3390/ijms17020189 10.3390/ijms17020189 PMC4783923 26848654

[90] Lewerenz J, Maher P (2015). Chronic glutamate toxicity in neurodegenerative diseases-what is the evidence?. Front Neurosci.

[91] Machado AK, Andreazza AC, da Silva TM, Boligon AA, do Nascimento V, Scola G, Duong A, Cadona FC, Ribeiro EE, da Cruz IB (2016) Neuroprotective effects of Acai (Euterpe oleracea Mart.) against rotenone in vitro exposure. Oxid Med Cell Longev 2016:8940850 10.1155/2016/8940850 10.1155/2016/8940850 PMC5066013 27781077

[92] Torma PD, Brasil AV, Carvalho AV, Jablonski A, Rabelo TK, Moreira JC, Gelain DP, Flores SH, Augusti PR, Rios AO (2017). Hydroethanolic extracts from different genotypes of acai (Euterpe oleracea) presented antioxidant potential and protected human neuron-like cells (SH-SY5Y). Food Chem.

[93] Xie C, Kang J, Li Z, Schauss AG, Badger TM, Nagarajan S, Wu T, Wu X (2012). The acai flavonoid velutin is a potent anti-inflammatory agent: blockade of LPS-mediated TNF-alpha and IL-6 production through inhibiting NF-kappaB activation and MAPK pathway. J Nutr Biochem.

